# Movement Therapy and Cognitive Function in Middle-Aged and Older Adults: A 10-Year Study

**DOI:** 10.1093/geroni/igaa057.1174

**Published:** 2020-12-16

**Authors:** Kallol Kumar Bhattacharyya, Hongdao Meng, Gizem Hueluer, Kathryn Hyer

**Affiliations:** University of South Florida, Tampa, Florida, United States

## Abstract

Cognitive function is an important component of healthy aging and physical activities have been shown to support late life cognitive function. However, it is unclear whether non-traditional physical activities provide additional benefits for cognitive function above and beyond traditional leisure physical activities. This study examines the associations between movement therapy and cognitive function in the US population. We used data from the waves 1, 2 and 3 (1995-2014) of the Midlife in the United States (MIDUS) study. MIDUS included a national probability sample of community-living adults aged 25-75 years old in 1995 (wave 1) and added the wave 2 cognitive functioning tests of executive function and episodic memory. We applied multivariate linear regression models to estimate the effect of movement therapy (wave 2) on the cognitive episodic memory and executive function (wave 3) while controlling the covariates (wave 2 sociodemographic factors, health, and cognitive function). A total of 2097 individuals aged 42-92 years (mean 64.4, sd 10.9, 55.6% women) were included in the analysis. Movement therapy was independently associated with better episodic memory (beta=0.117, p=0.02), but not with executive function (beta=0.039, p=0.14), after including control variables. The results suggest that movement therapy may be an effective non-pharmacological intervention to attenuate age-related cognitive decline in middle-aged and older adults. Future research should test whether these findings can be replicated in similar populations and if confirmed, interventions should incorporate a wider range of physical activities in community-living older adults with the goal of maintaining and improving physical and cognitive health.

